# Head-to-Head Comparison of Two Multi-Locus Sequence Typing (MLST) Schemes for Characterization of *Acinetobacter baumannii* Outbreak and Sporadic Isolates

**DOI:** 10.1371/journal.pone.0153014

**Published:** 2016-04-12

**Authors:** Franziska Tomaschek, Paul G. Higgins, Danuta Stefanik, Hilmar Wisplinghoff, Harald Seifert

**Affiliations:** 1 Institute for Medical Microbiology, Immunology and Hygiene, University of Cologne, Cologne, Germany; 2 German Centre for Infection Research (DZIF), partner site Cologne-Bonn, Cologne, Germany; Tianjin University, CHINA

## Abstract

To compare the two *Acinetobacter baumannii* multi-locus sequence typing (MLST) schemes and to assess their suitability to aid in outbreak analysis we investigated the molecular epidemiology of 99 *Acinetobacter baumannii* isolates representing outbreak-related and sporadic isolates from 24 hospitals in four different countries (Germany, Poland, Sweden, and Turkey). Pulsed-field gel electrophoresis (PFGE) was used as the reference method to determine the epidemiologic relatedness of isolates and compared to MLST using both the Oxford and Pasteur scheme. Rep-PCR was used to define international clonal lineages (IC). We identified 26 unique outbreak strains and 21 sporadic strains. The majority of outbreaks were associated with carbapenem-resistant *A*. *baumannii* harbouring oxacillinase OXA-23-like and corresponding to IC 2. Sequence types (STs) obtained from the Oxford scheme correlate well with PFGE patterns, while the STs of the Pasteur scheme are more in accordance with rep-PCR grouping, but neither one is mirroring completely the results of the comparator. On two occasions the Oxford scheme identified two different STs within a single outbreak where PFGE patterns had only one band difference. The CCs of both MLST schemes were able to define clonal clusters that were concordant with the ICs determined by rep-PCR. IC4 corresponds to the previously described CC15 Pasteur (= CC103 Oxford). It can be concluded that both MLST schemes are valuable tools for population-based studies. In addition, the higher discriminatory power of the Oxford scheme that compares with the resolution obtained with PFGE can often aid in outbreak analysis.

## Introduction

Members of the *Acinetobacter baumannii* group, i.e. *A*. *baumannii*, *A*. *nosocomialis*, *A*. *pittii*, and *A*. *seifertii* are a frequent cause of nosocomial infections, in particular ventilator-associated pneumonia, urinary tract, and bloodstream infections [1–3]. Patients in high-dependency care are mostly affected, and increasing multi-drug resistance worldwide is a cause of growing concern [4,5]. *A*. *baumannii* is known to be the most frequent *Acinetobacter* species isolated from patients in the ICU setting and it is known for its resistance to adverse environmental conditions, longevity on inanimate surfaces and propensity for epidemic spread [6]. *A*. *baumannii* is causing outbreaks of hospital infections around the world that are notoriously difficult to control.

A variety of molecular typing methods has been used for epidemiological characterization of *A*. *baumannii*, providing different levels of discrimination. The highly discriminative pulsed-field gel electrophoresis (PFGE) is still regarded as the method of choice when it comes to outbreak analysis [7,8]. Rep-PCR is a method that has been successfully applied to cluster *A*. *baumannii* isolates originating from different locations around the world [9]. It is particularly suited for a cost-effective analysis of a large number of isolates.

Another frequently used method is multi-locus sequence typing (MLST) which has been designed to study population structures of bacterial pathogens [10]. Two MLST schemes have been developed for *A*. *baumannii*. The first was introduced by Bartual and coworkers (Oxford scheme) [11] and a second scheme was developed at the Pasteur Institute (Pasteur scheme) [12,13]. Both schemes are working with seven housekeeping genes and have three genes in common.

Previous work has shown the presence of three distinct clonal lineages of *A*. *baumannii*, that because of their geographical distribution were designated European clones (EU) I, II and III [14,15]. When it became apparent that these lineages were distributed worldwide, they were termed international clonal lineages (IC), with IC1-IC3 corresponding to EU I-III [9,16]. To date we have identified eight ICs termed IC1-IC8 [9]. The majority of outbreaks reported around the world have been shown to involve MLST clonal complexes CC92/CC2 (Oxford/Pasteur), corresponding to IC2, and CC109/CC1 (Oxford/Pasteur), corresponding to IC1, respectively [7,14,17–19].

The aim of the present study was to compare the discriminatory power of the Oxford and the Pasteur MLST scheme and to evaluate whether MLST is able to provide additional information at the subspecies level that could aid in *A*. *baumannii* outbreak analysis, using PFGE as the reference method.

## Materials and Methods

### Bacterial isolates

Between 2006 and 2012, 298 *A*. *baumannii* isolates were submitted to our research laboratory by hospital epidemiologists from 24 hospitals in 4 different countries (17 hospitals in Germany, 5 in Turkey and one each in Poland and Sweden) to analyse their clonal relatedness and to confirm or refute the hypothesis that the isolates represented a hospital outbreak based on their epidemiological relatedness in space and time. One isolate per patient was included. Species identification of *A*. *baumannii* was confirmed using *gyrB* multiplex PCR as described previously [20,21]. Carbapenem MICs determined by VITEK2 (bioMérieux, Nürtingen, Germany) were confirmed by Etest (bioMérieux). OXA-multiplex PCR was used to detect *Acinetobacter* carbapenemase encoding *bla*_OXA_ genes as previously described [22,23]. The initial epidemiologic analysis of all isolates was performed by rep-PCR as described below. Of these, a total of 99 *A*. *baumannii* isolates were selected for further investigations based on their rep-PCR patterns (from each hospital, 2–5 isolates per given rep-PCR pattern and all isolates with aberrant patterns were included).

### PFGE

PFGE was performed as described previously, to compare isolates originating from one institution and to delineate outbreak-related and sporadic isolates [8]. The analysis of band patterns was performed visually and a difference of three bands or less was used to define close and of four to six bands difference to indicate distant epidemiological relatedness as suggested by Tenover et al. [24]. For the purpose of this study, two or more isolates from a given hospital and related in time and space with an identical or closely related PFGE pattern were considered to represent an outbreak. Pulsotypes were assigned capital letters; subtypes exhibiting one to three band differences were assigned numerals. No attempt was made to compare banding patterns of isolates across different hospitals.

### Rep-PCR

Epidemiological typing of all isolates was performed by rep-PCR [DiversiLab, bioMérieux] as described previously [9,16]. The analysis of rep-PCR patterns was carried out with the DiversiLab software, using the Pearson correlation. A similarity index of ≥ 98% and ≥ 95% was chosen to define a rep-PCR cluster of identical and related isolates, respectively [16]. The isolates were assigned to each of the eight ICs using our in-house library [9].

### MLST

MLST was performed using both the Oxford and Pasteur schemes as described previously [11,13], with minor modifications. Primers and PCR conditions are listed at http://pubmlst.org/abaumannii/ which now hosts both MLST schemes. The MLST sequences were uploaded to http://pubmlst.org/abaumannii/ to identify alleles and sequence types. Clonal complexes (CCs) were assigned by Burst and were defined as single-locus (SLVs) and double-locus variants (DLVs).

## Results

### PFGE

Based on PFGE pattern analysis, there were 78 outbreak-related isolates representing 26 distinct outbreaks termed OUT1-OUT26 (Table 1), while 21 isolates were considered sporadic and termed S01-S21. Among outbreak isolates, 4 outbreaks comprised 2 different subtypes whereas one outbreak comprised 6 different subtypes A1-A6.

**Table 1:** Epidemiological characterization of 26 outbreak strains using PFGE, rep-PCR, and MLST.

Strain/hospital^1^	PFGE-type/hospital^2^	IC^3^ (repPCR)	ST^4^		CC^5^	
			Oxford	Pasteur	Oxford	Pasteur
OUT01/H01	A/H01	2	436	2	92	2
OUT02/H01	B/H01	2	437	2	92	2
OUT03/H02	A/H02	1	439	1	109	1
OUT04/H03	A/H03	2	350	2	92	2
OUT05/H07	A/H07	2	436	2	92	2
OUT06/H08	A/H08	2	195	2	92	2
OUT07/H09	A/H09	2	208	2	92	2
OUT08/H10	A1-A3/H10	2	208	2	92	2
	A4-A6/H10	2	425	2	92	2
OUT09/H10	B1/H10	1	441	1	109	1
	B2/H10	1	441	1	109	1
OUT10/H11	A/H11	2	436	2	92	2
OUT11/H12	A/H12	2	208	2	92	2
OUT12/H13	A1/H13	2	208	2	92	2
	A2/H13	2	208	2	92	2
OUT13/H14	A1/H14	1	231	231	109	1
	A2/H14	1	442	231	109	1
OUT14/H16	A/H16	8	391	157	447	157
OUT15/H17	A/H17	2	195	2	92	2
OUT16/H18	A1/H18	2	557	2	92	2
	A2/H18	2	558	2	92	2
OUT17/H18	B/H18	2	448	2	92	2
OUT18/H19	A/H19	2	448	2	92	2
OUT19/H20	A/H20	2	452	2	92	2
OUT20/H21	A/H21	2	436	2	92	2
OUT21/H22	A/H22	2	195	2	92	2
OUT22/H22	B/H22	2	218	2	92	2
OUT23/H23	A/H23	2	218	2	92	2
OUT24/H23	B1/H23	7	229	25	110	25
	B2/H23	7	229	25	110	25
OUT25/H23	C/H23	2	441	1	109	1
OUT26/H24	A/H24	2	437	2	92	2

^1^Outbreak strains are numbered OUT01-OUT26; hospitals were named H01-H24; ^2^Capital letters indicate PFGE patterns that relate to a given hospital only, i.e. pulsotype A/H01 is different from pulsotype A/H02; numerals indicate PFGE subtypes

^3^IC, international clone

^4^ST, sequence type

^5^CC, clonal complex

### Rep-PCR analysis

Based on rep-PCR analysis, 72 of the isolates were assigned to IC2 and constituted the largest group (72.7%), followed by IC1 (15 isolates), IC7 (6 isolates), IC8 (3 isolates), and IC4 (2 isolates). One isolate did not cluster with any of the described international lineages. IC2 also comprised the largest number of distinct hospital outbreak strains, i.e. 20 of 26 outbreaks (76.9%) were caused by clonal dissemination of IC2 strains (Table 1). IC1 strains were implicated in four outbreaks, while one outbreak each involved IC7 and IC8 strains, respectively. Also among the sporadic isolates, IC2 represented the predominant clonal lineage, accounting for 10 isolates (47.6%), followed by IC1 (5 isolates, 23.8%), IC4 and IC7 (each 2 isolates, 9.5%), while one isolate represented IC8 and one was unrelated to previously described ICs.

### MLST analysis with the Oxford scheme

With the Oxford scheme 28 sequence types (ST), each containing 1–14 isolates were identified (Table 2). We were unable to assign a sequence type to two isolates belonging to IC1 and IC7, respectively (see below). All STs grouped into five CCs with the exception of ST499 being a singleton (Table 2). All IC1 isolates as determined by rep-PCR were CC109 composed of six STs differing in their *gpi* and in some cases also in their *gyrB* alleles. One IC1 isolate had IS*Aba1* inserted in the *gdhB* allele and we were therefore unable to assign a ST. All IC2 isolates corresponded to CC92 and were composed of 15 STs. Similar to CC109, all allelic variance was in *gyrB* and *gpi*. The two IC4 isolates correlated with CC103 and comprised the SLVs ST438 (*gpi*) and ST500 (*gpi*, *gyrB* and *gltA*). The six IC7 isolates correlated with CC110 and comprised the SLVs ST229 and ST440 (*gpi*) and one isolate with no assigned ST because of an IS*Aba125* insertion in the *gdhB* allele. The three IC8 isolates correlated with CC447 and were ST391 and ST447.

**Table 2:** International clones and corresponding CCs and STs of both MLST schemes based on the investigation of 99 *A*. *baumannii* outbreak and sporadic strains.

	CC		ST	
IC (repPCR)	Oxford	Pasteur	Oxford	Pasteur
IC1	109	1	231	1
			231	230
			231	231
			439	1
			441	1
			442	1
			442	231
			449	20
			498	81
			n.d.^1^	233
IC2	92	2	195	2
			208	2
			218	2
			281	2
			350	2
			425	2
			436	2
			437	2
			448	2
			450	2
			451	2
			452	2
			502	2
			557	2
			558	2
IC4	103	15	438	15
			500	232
IC7	110	25	229	25
			440	25
			n.d.^1^	25
IC8	447	157	391	157
			447	10
Unrelated	singleton	singleton	449	158

^1^ No ST determined (IS*Aba1* inserted in *gdhB*)

### MLST analysis with the Pasteur scheme

Using the Pasteur MLST scheme, the isolates belonged to 13 STs, which clustered in five CCs and one singleton (ST158) (Table 2). All IC1 isolates were CC1 which comprised six different STs whereas all IC2 isolates were CC2 represented by a single ST, ST2. The two IC4 isolates were assigned to CC15 comprising sequence types ST15 and ST232, whereas IC8 corresponded to CC157 encompassing two STs, ST 10 and ST157. Similar to the Oxford scheme the six IC7 isolates were all ST25 and belonged to CC25.

Compared to the Oxford scheme, within all isolates, there were fewer STs in the Pasteur scheme (Table 2). However, this is mainly due to the fact, that isolates representing IC2 were assigned 15 different STs within the Oxford and only one ST within the Pasteur scheme. In contrast, IC1 had six different STs assigned in both schemes. Among these, ST231 (Oxford) had three STs (Pasteur), i.e. ST1, ST230, and ST231, whereas ST442 (Oxford) had 2 STs (Pasteur) assigned, i.e. ST1 and ST231. Conversely, ST1 (Pasteur) had four STs (Oxford) assigned, namely ST231, ST439, ST441, and ST442. The remaining ICs, IC4, IC7 and IC8 were represented by only a few isolates each and were assigned similar numbers of STs in both schemes. Although there was no complete correlation between STs from both schemes, CCs of the Oxford and the Pasteur scheme were corresponding to each other. Each IC identified using rep-PCR had a corresponding CC using MLST, and the CCs of both schemes were concordant (Table 2).

### Fine Typing

Obvious differences in resolution of strains that might be used for outbreak delineation were only observed among IC2 isolates where the Pasteur scheme did not provide resolution below the CC level whereas with the Oxford scheme 15 different ST were distinguished among our isolates (Table 2). We have several examples where these variations in STs did aid in outbreak analysis. In hospital H01 we observed two different outbreak strains OUT1 and OUT2 based on PFGE patterns A and B that could also be resolved by MLST using the Oxford scheme with two different STs 436 and 437 (Table 3). Similar observations were made in hospital H18 with two outbreak strains OUT16 and OUT17 involving STs 557/558 and 448 as well as in hospital H22 with two outbreaks OUT21 and OUT22 involving STs 195 and 218; all these strains were assigned a single ST in the Pasteur scheme, i.e. ST2. Also, there were several sporadic isolates recovered during the course of an outbreak that could be distinguished from the outbreak strain based on their Oxford ST whereas this was not possible when isolates were typed using the Pasteur scheme (data not shown).

**Table 3:** Outbreak isolates from six hospitals analysed with PFGE and both MLST schemes.

				MLST Oxford Scheme									MLST Pasteur Scheme								
Strain/hospital^1^	IsolateNo.	PFGE type^2^	IC^3^	*gltA*	*gyrB*	*gdhB*	*recA*	*cpn60*	*gpi*	*rpoD*	ST^4^	CC^5^	*cpn60*	*fusA*	*gltA*	*pyrG*	*recA*	*rplB*	*rpoD*	ST^4^	CC^5^
OUT01/H01	56	A	IC2	1	3	3	2	2	103	3	436	92	2	2	2	2	2	2	2	2	2
OUT02/H01	108	B	IC2	1	12	3	2	2	97	3	437	92	2	2	2	2	2	2	2	2	2
OUT04/H03	2929	A	IC2	1	12	3	2	2	102	3	350	92	2	2	2	2	2	2	2	2	2
SPOR/H03	5247	B	IC2	1	3	3	2	2	97	3	208	92	2	2	2	2	2	2	2	2	2
OUT08/H10	51	A1	IC2	1	3	3	2	2	97	3	208	92	2	2	2	2	2	2	2	2	2
OUT08/H10	208	A2	IC2	1	3	3	2	2	97	3	208	92	2	2	2	2	2	2	2	2	2
OUT08/H10	102	A3	IC2	1	3	3	2	2	100	3	425	92	2	2	2	2	2	2	2	2	2
OUT08/H10	103	A4	IC2	1	3	3	2	2	100	3	425	92	2	2	2	2	2	2	2	2	2
OUT16/H18	7182	A1	IC2	1	38	3	2	2	66	3	557	92	2	2	2	2	2	2	2	2	2
OUT16/H18	4278	A2	IC2	1	17	3	2	2	16	3	558	92	2	2	2	2	2	2	2	2	2
OUT17/H18	2502	B	IC2	1	15	3	2	2	98	3	448	92	2	2	2	2	2	2	2	2	2
OUT21/H22	3701	A	IC2	1	3	3	2	2	96	3	195	92	2	2	2	2	2	2	2	2	2
OUT22/H22	3463	B	IC2	1	3	3	2	2	102	3	218	92	2	2	2	2	2	2	2	2	2
OUT13/H14	G112	A1	IC1	10	12	4	11	4	98	5	231	109	1	1	1	1	5	1	4	231	1
OUT13/H14	G131	A2	IC1	10	12	4	11	4	99	5	442	109	1	1	1	1	5	1	4	231	1

^1^Outbreak strains are numbered OUT01-OUT26; hospitals are named H01-H24

^2^PFGE type designation is restricted to one hospital

^3^IC, international clone

^4^ST, sequence type

^5^CC, clonal complex

Nevertheless, the ST of the Oxford scheme can probably not be used as an alternative to PFGE. As an example, we observed one outbreak in hospital H14 where two isolates had very similar PFGE patterns with one band difference but showed two different STs, ST231 and ST442, a SLV within the *gpi* locus of the Oxford scheme, and only one ST (ST231) in the Pasteur scheme (OUT13, Table 3). In hospital H10, isolates involved in an outbreak extending over a period of 20 months (OUT08) comprised 6 closely related subtypes determined by PFGE but two different STs, ST208 and ST425, again a SLV within the *gpi* locus of the Oxford scheme, and one ST (ST2) in the Pasteur scheme. However, subtle differences in PFGE patterns were not always reflected by differences in Oxford MLST types (OUT09, OUT12, and OUT24, Table 2).

### Carbapenem resistance mechanisms

In total, 84 of 99 isolates were carbapenem-resistant (84.8%, data not shown). Oxa-23-like accounted for 70 isolates, followed by Oxa-58-like (11 isolates; 2 of these had imipenem MICs ≤8mg/L), and Oxa-24-like (2 isolates). Another 4 carbapenem-resistant isolates had IS*Aba1* upstream of the intrinsic Oxa_-51-like_ but no acquired oxacillinase. Among outbreak strains, 18 had Oxa-23-like, 3 had Oxa-58-like and one involved a carbapenem-resistant strain with IS*Aba1* upstream of Oxa_-51-like_, whereas 5 outbreaks involved strains with and without an acquired oxacillinase suggesting acquisition of Oxa-23-like and Oxa-58-like during the course of the outbreak (data not shown).

## Discussion

The number of outbreaks involving *A*. *baumannii* has been rapidly increasing over the past decades and multi-drug resistance is limiting available treatment options [1,4,6,7]. Molecular epidemiological investigation of bacteria is one of the pillars that trigger the institution of targeted infection control measures, which are required to eliminate *A*. *baumannii* and to prevent further spread. PFGE is suited for outbreak analysis and is well established for fine-scale typing in local epidemiological studies [7,8,17]. In the field of longitudinal epidemiological studies, the automated rep-PCR with its internet database and web-tools is an approved method that can also be used for outbreak analysis but its discriminatory power is inferior to PFGE [9,16,25].

MLST is often seen as a complementary typing method to PFGE, with a focus on clustering isolates on a global basis [12,13,25,26]. Theoretically, MLST could be used for both, global epidemiological studies and small-scale outbreak analysis. However, there are some unresolved questions, including whether the criteria for the selection of housekeeping genes are sufficiently reliable to reveal the population structure and how many loci there are required to obtain a reliable typing scheme. A study of Woo et al showed, that the achieved discriminatory power of six gene loci was the same as the one achieved by sequencing seven loci, with either *gdhB* or *recA* being left out in the Oxford scheme [27]. Furthermore, without *gpi* and *gyrB*, the five remaining genes provide sufficient discriminatory power to separate the major ICs [28]. In this study, the CCs of both MLST schemes showed very high similarity to the ICs and to each other, with each IC being matched by one CC as shown in previous studies [13,26]. Coincidently, IC4 strains as determined by rep-PCR were found to be CC103 [Oxford] and CC15 [Pasteur] which Diancourt et al recognized as, but did formally name the fourth international clone [13].

The number of STs was much higher in the Oxford scheme, especially within the IC2 group. This could indicate a higher discriminatory power of the Oxford compared to the Pasteur scheme, and in addition brings up the question if it could be even used for fine typing. We had a number of examples that seem to corroborate this hypothesis, with a ST in the Oxford scheme and PFGE showing the same subdivision of strains, while the Pasteur scheme did not differentiate between different outbreak and some sporadic strains within a given epidemiological setting. However, there were two examples to the contrary where it even seemed that MLST in the Oxford scheme showed differences among outbreak strains that were not mirrored by PFGE. It has been suggested that the high variability of the *gpi* and *gyrB* genes of the Oxford scheme might be due to recombination [28,29].

Furthermore, it has to be mentioned that the diversity of the STs in this study varied depending on the respective IC, although we do not know the cause of it. Within our IC2 strains, there was only one ST in the Pasteur scheme facing 15 STs provided by the Oxford scheme. In the group of the IC1 strains on the other hand, the diversity was comparable with six different STs in each MLST scheme.

In conclusion, both MLST schemes are dependable tools for global epidemiological studies, regarding the fact that *A*. *baumannii* is clonal in nature [11,13,15]. Further resolution at the strain level that is based mainly on the *gpi* and *gyrB* gene loci in the Oxford scheme often gives additional information that at least among IC2 isolates may aid in local outbreak analysis as well as to establish epidemiological links between outbreaks. However, MLST using the Oxford scheme is still less discriminative than PFGE but occasionally may be too diverse as to be able to fully substitute PFGE. It remains to be determined how MLST will be used in the future when these data will be easily extractable from next generation sequencing data.
